# Experiences of U.S. frontline physicians during the COVID-19 pandemic: a qualitative study

**DOI:** 10.1186/s13690-025-01609-0

**Published:** 2025-05-07

**Authors:** Robin L. Whitney, Melissa Gosdin, Sabrina F. Loureiro, Marykate Miller, Joy Melnikow, Richard L. Kravitz

**Affiliations:** 1 Betty Irene Moore School of Nursing, University of California Davis, 2570 48th Street, Sacramento, CA 95817 USA; 2 The Valley Foundation School of Nursing, San José State University, San José, USA; 3 Center for Healthcare Policy and Research, University of California, Davis, Sacramento, USA; 4 NYU School of Global Public Health, New York, USA; 5 Department of Family and Community Medicine, University of California, Davis, Sacramento, USA; 6 Department of Internal Medicine, University of California, Davis, Sacramento, USA

**Keywords:** COVID-19, Pandemic response, Physician wellness, Physician burnout

## Abstract

**Background:**

The COVID-19 pandemic caused profound and rapid changes in patient care and healthcare system organization. There is a compelling need for insight into the challenges that confronted physicians during the early phase of the pandemic to identify successful adaptations and strategies that minimize disruption to patient care and protect clinician wellbeing. The purpose of this study was to understand physicians’ lived experiences of providing patient care during the early COVID-19 pandemic.

**Methods:**

This qualitative, descriptive study used a thematic analysis approach. The sample included 17 physicians from five specialties with direct care experience of COVID-19 patients (infectious disease, primary care, emergency medicine, critical care, and hospitalists). Participants were identified through snowball sampling. Data were collected through focus groups and interviews in May and June 2020 and analyzed with an inductive and deductive approach using thematic analysis.

**Results:**

Three overarching themes relating to patient care delivery during the ongoing COVID-19 pandemic were identified: facilitators, barriers, and acute stressors. Facilitator subthemes included: organizational logistical and operational support, organizational support for self-care and wellness, and peer and family support/debriefing. Barrier subthemes included: lack of clear and consistent governmental guidelines and organizational support, uncertainty resulting from poor communication or lack of information, and interpersonal barriers to physician self-care and wellbeing. Stressor subthemes included: concern about exposure, feeling unprepared, and anticipating the worst.

**Conclusions:**

Physicians reported that both patient care and their own wellbeing were greatly impacted by organizational and systems level facilitators and barriers. Findings from this study can inform the creation of best practices, tools, and strategies that can assist with future emergency preparedness and pandemic response planning efforts.

**Supplementary Information:**

The online version contains supplementary material available at 10.1186/s13690-025-01609-0.

**Table:** 

**Text box 1. Contributions to the literature**
• The COVID-19 pandemic disrupted healthcare operations worldwide, creating care delivery challenges and stressful healthcare work environments, particularly during its early stages.
• Frontline physicians who provided direct patient care and assumed healthcare leadership roles during the early pandemic are uniquely positioned to provide valuable insights on the challenges and opportunities healthcare workers experienced.
• Qualitative exploration of physicians’ early-pandemic experiences can shed light on barriers and facilitators to patient care and physician wellbeing and improve responses to future crises.

## Background

The COVID-19 pandemic had sudden and disruptive impacts on healthcare delivery worldwide. COVID-19’s transmissibility and rapid spread required a swift response from all tiers of the healthcare delivery system. Changes in scientific understanding, public health recommendations, and patient care policies prompted systems to adjust, often in real time. Frontline healthcare workers have borne the brunt of the pandemic’s impacts, and pandemic-related stressors continue to affect the wellbeing of the healthcare workforce [1]. A close examination of healthcare workers’ responses and adaptations to the COVID-19 pandemic, particularly during its early phases, is needed to understand the potential long-term impacts and improve responses to future crises. Physicians, as frontline workers and healthcare system leaders, are in an ideal position to distinguish successful practices, identify gaps, and advocate for policies to support healthcare workers.

Several recent studies have reported on the COVID-19 pandemic’s impact on healthcare workers, including substantial work-related stress, emotional exhaustion, and disruptions to work-life balance [2–5]. During the pandemic’s earliest phases, a large proportion of frontline healthcare workers experienced acute stress, depressive symptoms, or anxiety, largely driven by uncertainty around treatment, testing, and infection control guidelines, as well as shortages of personal protective equipment (PPE) [6]. Internationally, policy responses to the COVID-19 pandemic varied widely, yielding different incidence and mortality rates across settings, especially in the early pandemic before widespread vaccine availability [7–9]. However, despite this variation, many stressful experiences that impacted frontline workers were similar across settings [9]. 

Personal risk related to inadequate PPE access was an especially prominent concern for frontline physicians who interacted with patients infected with COVID-19 during the early waves. Although improvements in treatment and the availability of mRNA vaccines mitigated the severity of later waves, in its early phases there was substantial COVID-19-related morbidity and mortality among healthcare workers [10, 11]. Indeed, Kiang et al. reported that excess mortality among physicians averaged 50–80 excess deaths per month during the pandemic prior to the availability of the vaccine [12]. 

In addition to creating fear and distress, infections and exposures exacerbated already existing physician staffing shortages as COVID-19 admissions surged. One hospital in the in New York epicenter of the first wave reported that nearly 25% of its physician residents were unavailable by mid-March 2020 due to illness or quarantine protocols [13]. In combination with the influx of COVID-19 patients, these shortages necessitated sudden and drastic changes to routine care, such as cancelling elective surgeries and redeploying physicians and residents specializing in surgery, anesthesiology, and cardiology to assist internal medicine services [13, 14]. 

These stressors and organizational responses to them varied across settings (e.g., rural versus urban areas) and types of organizations (e.g., large integrated health systems vs. community hospitals vs. private outpatient practices) [15]. While some early-pandemic stressors have resolved, others have persisted, adding to already high levels of burnout [16, 17]. Results from a national survey of US physicians found a sharp increase in the percentage of physicians who experienced symptoms of burnout between 2020 and 2021 [17]. Experiencing burnout (specifically emotional exhaustion and depersonalization) was associated with intention to reduce clinical work hours, which was reported by only 20% of physicians in 2014 and increased to 40% 2021 [18]. To improve our ability to respond to future pandemics and ensure a robust and healthy physician workforce, we must learn from their experiences throughout the pandemic, including what adaptations were successful and should be disseminated to support the nation’s response to future public health emergencies.

Several studies have reported on COVID-19 pandemic-related experiences and impacts on the physician workforce, specifically [3, 5, 19–26]. However, relatively few have examined the experiences of physicians working within the United States healthcare system [19, 22, 23]. Buchbinder et al. and Ferber et al. examined factors contributing to stress and burnout among physicians during the COVID-19 pandemic, but conducted their studies during later stages of the pandemic, from November-December 2020 [22] and February-October 2021 [19]. Frank et al. surveyed U.S. physicians somewhat earlier in the pandemic, August 2020, but focused on the pandemic’s impact on work-family conflict and only included physicians who identified as parents in their sample [23]. 

## Methods

### Design

This qualitative descriptive research was part of a series of mixed methods studies examining physicians’ experiences and well-being during the 2020 COVID-19 pandemic [1]. Physicians from across the United States participated in either an online focus group or individual interview each lasting approximately an hour. This study was considered exempt from review by the University of California, Davis institutional review board. Participants gave verbal consent before participating. We have reported our findings in alignment with the standards for reporting qualitative research (SRQR) [27]. 

### Study participants

A snowball sample [28] of 17 physicians were identified through professional contacts of the PI (J.M.) and asked to participate. Physicians were included from diverse geographic regions within the U.S. and across five specialties: infectious disease, primary care, emergency medicine, critical care, and hospitalists.

### Approach

In May and June of 2020, a total of 8 interviews and 3 focus groups took place virtually, each conducted by a trained moderator (either M.G., M.M., D.R., or S.L.), who asked participants open-ended questions from a semi structured interview guide designed with the dual purpose of exploring clinicians’ early pandemic experiences and eliciting formative feedback to develop a survey for the quantitative aspect of the larger project (Appendix Table A1). Topics included professional and personal experiences occurring during the early COVID-19 pandemic and acute stressors from the viewpoint of practicing physicians. Specific probes were used to elicit information about changes to medical practice, social/emotional support and use of telehealth. Additionally, we collected basic descriptive information for each participant regarding their gender, specialty area, geographic region, urban vs. rural status, and years of experience in practice. Interviews were recorded and transcribed verbatim.

Thematic analysis was conducted using an open coding and constant comparison approach [29]. Investigator triangulation among four independent coders (M.G., M.M., D.R., & S.L.) was utilized to ensure rigor. Within the coding team, two pairs were formed, each assigned to review and code a set of transcripts and develop a codebook. Codes were created both inductively, (emergent codes) and deductively (priori codes) [30]. After each researcher independently reviewed and coded the transcripts, pairs met to debrief and reach intercoder agreement. Any disagreements were discussed until a consensus was reached. This process was then repeated within the larger team. Themes and subthemes were produced and agreed upon by all coders by collapsing individual codes and creating categories. Themes and subthemes are presented with illustrative quotes edited lightly for readability (e.g., removing filler words such as “um”). The interviews were conducted by a combination of non-clinical research staff and academic primary care physicians who were concurrently experiencing the effects of the pandemic as frontline physicians. These shared experiences may have influenced interpretation of participant comments and ideas for probes.

## Results

### Sample

A total of seventeen physicians across five specialties including primary care (*n* = 5), emergency medicine (*n* = 5), critical care (*n* = 2), hospital medicine (*n* = 3) and infectious disease (*n* = 2) participated in this study. Of the seventeen participants, 58% identified as male, 88% practiced in an urban setting, and a majority reported being early- to mid-career. Participants practiced in 1 of 5 regions of the U.S. including West (*n* = 6) Midwest (*n* = 5), Northeast (*n* = 3), Southwest (*n* = 1) and Southeast (*n* = 2) (Table 1).

**Table 1:** Characteristics of study participants: physicians during the early COVID-19 Pandemic in the United States (*N* = 17)

Characteristics	No. (%)
Sex	
Female (vs. Male)	7 (41.2)
Specialty	
Primary Care	5 (29.4)
Hospitalist	3 (17.7)
Emergency Medicine	5 (29.4)
Infectious Disease	2 (11.8)
Critical Care	2 (11.8)
Clinical Site	
Urban (vs. Suburban)	15 (88.2)
Region	
West	6 (35.3)
Midwest	5 (29.4)
Northeast	3 (17.7)
Southwest	1 (5.9)
Southeast	2 (11.8)
Time since completing clinical training, in years	
0–5	4 (23.5)
6–10	4 (23.5)
11–20	6 (35.3)
21–30	1 (5.9)
31–40	2 (11.8)

### Themes

We organized our findings into three overarching themes: (1) facilitators of patient care and physician wellbeing; (2) barriers to patient care and physician wellbeing and (3) perceived stressors, each with subthemes relating to systemic, organizational and/or physician-level factors. Physician insights regarding facilitators and barriers to patient care and wellbeing are summarized in Table 2 and additional quotes are available in the supplementary appendix Table A2.

**Table 2:** Barriers & Facilitators to Patient Care and Physician Wellbeing During the Early COVID-19 Pandemic in the United States

	Facilitators	Barriers
**Patient Care**	• Clear, organized policies & protocols • Clear, frequent organizational communication • Adequate PPE • Telehealth	• Confusing or contradictory guidelines • Uncertainty about diagnosis and treatment • Reimbursement uncertainties • Lack of recovery/rehab services • Difficulty planning care delivery in light of uncertainty • Misalignment between organizational/government resources and frontline care needs
**Physician Wellbeing**	• Wellness programs • “Bottom-up” peer support • Family support	• Need to postpone critical non-COVID-related work • Worry about exposure • Pressure to protect family and staff • Worry about the future • Balancing home and work demands

### Theme 1: facilitators of patient care and physician wellbeing

Facilitators included those factors that promoted physicians’ ability either to provide effective care or to support their own wellbeing during the early pandemic.

#### Subtheme 1 A: organizational leadership and operational support

Leadership and operational support from the organization or larger health system played an important role in facilitating physicians’ ability to provide care during the COVID-19 pandemic. Participants across specialties described ways that they felt supported by their organizations. One primary care physician highlighted the way in which organizational leadership’s initiative in developing proactive PPE protocols made him feel protected: *“They fitted us for N-95s*,* and they used the hospital screening protocols and all that good stuff right from the outset*,* so that definitely felt safer.”* A critical care physician added:

*Fortunately*,* the hospital… leadership had actually done quite good with*,* planning in advance in terms of making resources available as well as in terms of actually opening a field hospital in order to really manage the patient population and I think similar adjustments were being done even in the ICU setting.*

Resource sharing as a form of organizational support was also viewed by several participants as a facilitator to patient care delivery. Sharing hospital resources included bringing in support staff. For example, adding additional physicians from various specialties assisted hospital and emergency care providers with the heavy patient load. An emergency room physician observed:

*The hospital did a lot of work to bring in other people to the Emergency Department so that we all weren’t working five days a week…I was working with Urologists and Ophthalmologists who had come in and we would train them for two shifts and then they would be attendings and second attendings*.

Similarly, many providers reported that leveraging available telehealth technology strengthened their capacity to treat and care for patients, particularly those from underserved communities. One primary care physician explained how pivoting to telehealth appointments not only helped them adjust to the new normal of the pandemic, but also offered a potential solution to improve care for their patient population going forward:

*We have issues around transportation…we have a vulnerable population. We also have a lot of construction going on and lack of parking*,* so [with telehealth appointments] our no-show rates had plummeted. When people were actually scheduling these virtual or telephone visits*,* they were more likely to keep them because*,* one*,* they have nothing else to do*,* they’re at home*,* and*,* two*,* they don’t have the challenges of getting to the clinic.*

To facilitate successful adaptations to the rapidly changing situation, flexibility on the part of hospitals and regulators was essential. One emergency physician described how their facility was able to work around existing regulations and “red tape” that typically slow the pace of change in healthcare:

*They have spun up telemedicine programs with an amazing degree of innovation. They’ve ignored requirements and regulations that previously would kind of bind them. And*,* fortunately*,* CMS has issued waivers and the Joint Commission has stopped inspections. And so hospitals are more willing to innovate and do things.*

#### Subtheme 1B: organizational protection of physician self-care and wellness

Participants appreciated when their healthcare system took actions that made protected workers from the brunt of pandemic’s impact. For example, several participants appreciated formal wellness support offered by healthcare organizations. One emergency physician highlighted how an established institutional wellness program in place before the pandemic allowed for physician access to video counseling sessions: *“I think our program in particular has a very strong wellness program and so they were very active in rolling out a lot of immediate issues that they made. We all had access to free video counseling.”*

Some organizations created new support services as a response to the pandemic. For example, one hospitalist physician explained that simply creating structured opportunities for frontline staff to debrief regularly was helpful: “*The institution has been very supportive…there’s a debriefing that*,* a system wide debriefing as well as a division level debriefing that has occurred and… and overall*,* from a professional perspective*,* it’s been a very supportive environment.”*

Wellness support was also sometimes provided by professional organizations, allowing access to physicians whose organizations did not offer such supports or who might wish for more anonymity. An emergency room physician explained:

*ACEP*,* the American College of Emergency Physicians that I also work with has also put together a video diary or testimonial that is private that allows people to discuss their*,* their feelings*,* their anxieties*,* their lessons learned that they would like to share*,* but in a way that’s private and not put out on social media*.

#### Subtheme 1 C: peer and family support/debriefing

Peer and family support were identified as coping mechanisms often used by physicians during the pandemic. Peer support was especially important as it allowed participants to connect with other physicians who were experiencing very similar circumstances while buffering stress. Organized, virtual social events with colleagues enabled some physicians to better cope with COVID-19 induced stress. An emergency medicine physician explained the benefits of connecting and debriefing with their peers: “*I find the most relief and solace when we can get together for a virtual happy hour with others in our group. The question is how can we do a better job at helping prepare and deal with pandemics? The most benefit is probably in doing more peer counseling.”*

A critical care physician expressed the benefit of talking with colleagues. *“In terms of dealing with it…I just try to talk it out with another colleague and let it go by”* while an emergency medicine physician elaborated on the impact of providing and receiving such support: *“I think where we turn to that’s healthy is turning to our peers for some peer support. And equipping our peers to better deal with that*,* I think*,* is really important.”*

A primary care physician also commented on the importance of connecting with others who are experiencing similar stressors:

*We [my fellow faculty] spend a lot of time together…we’re actually spending more time together*,* masked up and 10 feet apart*,* we talk through our office walls often. But that’s been very helpful to know that there’s a cadre of others who are going through the same things*,* both at work and at home.*

The comfort found in shared experiences also provided a feeling of connection to clinicians of the past. One primary care physician described how reading accounts of previous pandemics or disasters provided reassurance that there is some degree of predictability in the trajectory and experience of this type of event, despite the feeling of uncertainty and chaos

*What’s been really helpful to me personally is to know that these… this cycle or this path of milestones that occurs during disasters has been described before in the literature and actually what I’m going through is what is expected*,* which is this curve of having a lot of optimism in the beginning and almost like a heroicism. And then followed by a period of prolonged kind of disillusionment as you’re trying to work through all the workarounds.*

In addition to peer support, a primary care physician expressed how family support can help buffer stress. *“I think the main things that are helping me cope are good*,* family connections. So*,* having*,* and maintaining really close relationships and open communication with my immediate family.”*

### Theme #2 barriers to patient care and physician wellbeing

#### Subtheme 2 A: bureaucratic difficulties, lack of standardization in public health agency guidelines and organizational support

Participants described how a lack of clear and consistent governmental guidelines and inadequate organizational support created barriers to providing patient care during the COVID-19 pandemic. An emergency medicine physician commented on the inadequate leadership:

*There was definitely a very noticeable lack in coordination in leadership. I mean I think obviously on the federal level. But even on a local level it was*,* it was challenging. I work for the largest health system in the city and we have about 11 different hospitals across the city. And even within our health system*,* the burden of disease and the onset of this*,* the onslaught of patients were very different*.

The differences in resources and needs between regions and facilities made it challenging to standardize solutions. In some instances, well-intended policies implemented to support frontline providers did not address their needs. One hospitalist shared his frustration with misalignments between organizational policies and decisions and actual patient care needs on the front lines:

*It is very hard because what you would like to do and what policy would allow us to do are very different. Even when the military stepped in and started directing field hospitals*,* the purpose of the field hospitals was never to offload COVID patients from any hospital in the entire county…they were intended for non-COVID patients. But we were seeing a dramatic decline in non-COVID hospitalizations around the same time that COVID was booming around the country*.

Several participants provided examples of how telehealth was both a barrier to patient care and a facilitator depending on the context. Some physicians believed that telehealth could be strengthened at their organization if connectivity problems and billing issues were addressed. An emergency medicine physician explained:

*…Until recently we couldn’t charge for telemedicine visits…there’s very limited things that you can bill for telehealth. And so*,* if people couldn’t get compensation from it*,* they were much less likely to implement it. But now*,* at least from the COVID perspective*,* we’ve had an emergency thing put in at least temporarily.*

Barriers to telehealth were also related to infrastructure, especially in rural and underserved areas. The same emergency physician clarified that: *“in many rural areas of Texas where I am*,* we’ve talked about trying to help our rural hospitals that are failing*,* but they just don’t have the internet infrastructure in place to be able to even do telemedicine in a lot of places.”*

As organizations navigated rapid changes in care delivery, structural barriers sometimes limited the ability to adapt nimbly to evolving patient care needs. A primary care doctor described the financial pressures involved in decision-making around shifting to telehealth:

*So for example*,* my practice in the beginning said*,* “We don’t care if we get paid or not*,* we’re part of a large institution. We have a very vulnerable population. Many of them will not have the ability to use video or just not have the data minutes to do that. If we were a private practice and before the Medicare retroactively allowed for billing for the telephone visits*,* we would have had a very different conversation around how to keep potentially volumes either up or… and that was not an issue for us*.

Similarly, a critical care physician described how standard practices and reimbursement models did not account for patient needs beyond the acute phase of care:

*We don’t have a capacity to handle this many patients for the recovery phase for the rehab and so on. So*,* that’s also another external factor. Either insurance is going to change the reimbursement*,* or a health care policy have to give them more support for rehab to accept these patients so that they can get the care to recover*,* because I do believe the reason we are doing- providing this level of care is for them to recover and not to*,* just to prevent the deaths… I feel like system is failing because we don’t have a support for the recovery phase. We got a lot of support for the acute phase.*

#### Subtheme 2B: uncertainty resulting from poor communication or lack of preparedness

Several primary care physicians shared how uncertainty resulting from a lack of information created barriers to care delivery and negatively impacted their wellbeing.

*The way that information was communicated or has been communicated or even currently is communicated through the usual channels was altered because our understanding of the virus changed so much daily. So that was frustrating*,* but also actually pretty scary…*,* but what has happened and is continuing to happen is that we’re getting our sources of information from so many different places that we usually wouldn’t.*

Another primary care physician stated: “*I think it’s scattered. It feels like we don’t know the principles behind what we need to be doing and- and I think because of that it’s hard to figure out what we should be doing and figure out what our organizations should be doing to support us.”*

Physicians also described how rapidly changing information creates an uncertain future that can impede the clinical workflow while creating distrust for many healthcare providers. A primary care physician described the struggle to get current and reliable information during this phase of the pandemic:

*So*,* for me to get information on COVID from the New York Times would be very unusual but having the pressure of trying to get as much information as possible*,* not necessarily trusting the source of information and then having to answer questions from patients from information that had not really been vetted in a way that typical information would have been vetted. In this case the best I could do might be the CDC website which was behind and also I’d say the information that I was receiving as a physician was colored or influenced by the availability of the [COVID] test. That was a problem.*

#### Subtheme 2 C: interpersonal barriers to physician wellbeing

As physicians navigated many clinical challenges related to patient care, their personal and professional lives were also impacted by the pandemic. A primary care physician with school-aged children described how the pandemic has become more challenging over time due to the impact on in-person schooling and childcare:

*I would say that the first few weeks*,* I think we were all… I’ll just speak for myself*,* was probably more optimistic and felt that it was likely a time-limited change in practice. But I think as the weeks have progressed*,* it’s actually gotten harder*,* not medically but actually personally has gotten harder to balance working full time*,* managing a household with children*,* school-age children*.

Another primary care physician described the stress of worrying about the pandemic’s impact on professional goals and milestones: *“I think the other piece that people who are building things*,* like let’s say QI initiatives in their clinic or research programs is this idea that you have dedicated your career or your professional life to projects that now have become derailed.”*

An emergency medicine physician (and others) also noted that, despite the many stressors they were facing, there were barriers to seeking help to manage stress and address mental health concerns:

*I think there’s still a lot of reluctance amongst physicians for both good and bad reasons to seek formal help…we all hear that one crazy case of that person who tried to do the right thing and*,* and address their depression and stress and instead things went haywire and they lost their license when they got reported to the medical board*,* right? So I think there’s always fear about that issue*.

### Theme #3: acute stressors

Physicians, regardless of specialty, reported acute stressors that made their jobs difficult such as concern about exposing either themselves or their family to COVID-19, not being able to protect the staff that worked with them, feeling unprepared to answer questions and adequately treat patients and anticipating the worst due to misinformation and/or information overload.

#### Subtheme 3 A: concern about exposure

A major concern for physicians practicing during the early months of the COVID-19 pandemic was the fear of exposing others to the virus. The perceived responsibility to protect others often resulted in guilt and frustration. A primary care physician explained:

*…having a sense of responsibility to the people who work with us. So*,* our MAs*,* our nurses*,* our support staff and the idea of not being able to protect them in the way that we feel as doctors should be happening… and I know that this employee is pregnant or has asthma or this employee has a heart condition and I am supposed to tell them all to keep coming to work… that sense of going against what clinically you feel is right [in contrast with]what the health system needs to function.*

Some participants recognized the need for self-care as a means of caring for others. A critical care physician explained:

*I tried to go outside… to the park*,* to walk around before I go to ICU. I do start preparing probably two days prior. I go to the ICU just to keep myself in correct mode because I have to take care of myself*,* but I also have to take care of a resident and the nursing staff and patient and patient families. So*,* I really have to keep myself neutral and strong…so that’s a lot.*

In addition to feeling responsibility for their staff, many physicians expressed concern for their families. A primary care physician expressed particular concern for a partner with an autoimmune disease.

*my [spouse] has had rheumatoid arthritis for 40 years. I told the team*,* I am happy to do inpatient work. I’m happy to do outpatient work. I’m going to choose not to do the COVID team because I… while I think I would do okay even if I got it*,* I don’t need my wife to get it.*

#### Subtheme 3B: feeling unprepared for new clinical challenges

All participants reported feeling unprepared for COVID-19, regardless of the organization they worked for. Feelings of unpreparedness due to lack of treatments, rapidly changing guidelines, and lack of data induced stress for many. According to another primary care physician, *“we honestly didn’t know how to answer a lot of the questions in the beginning*,* so that was a great…a big source of stress was that if a patient emailed me*,* I wouldn’t necessarily know even how to answer them.”*

An emergency medicine physician expressed that not having answers during the pandemic differed from the uncertainty he was used to in the emergency department: “*As an ER doctor I am accustomed to making decisions with incomplete information but in this case with the stakes being particularly high it’s very stressful to be looked to as the person who has the answer when no answers exist.”*

A primary care physician described how not knowing how to answer questions from patients and staff produced anxiety for many physicians: *“I would say there’s a lot of uncertainty about what to recommend to people. I feel like things are constantly changing. I think anxiety is a little bit higher… I would go back and say that uncertainty is related both to clinical things as well as workflow and administrative tasks.”*

#### Subtheme 3 C: anticipating the worst

Physicians also discussed the impact that waiting for things to get a lot worse had on their mental and emotional well-being. An emergency physician described that: “*I kind of always felt like*,* I don’t know if anyone’s played a sport but*,* you get kind of nervous before a match and then the match gets delayed. And gets delayed again and you’re just in a state of nerves all the time… you just want to get it done.”*

The anxiety of anticipation was worsened when the pandemic did not follow the expected course. An emergency room physician described the feeling of bracing for a surge only to find that the ED was actually slower than usual:

*From an emergency department perspective*,* I think there was a preparation for an onslaught. We’re so used to disasters being presented as this surge of sudden onrushes of patients. So*,* we were really preparing for that at least across the West Coast and [at our] sites. And really what we found was more of this slow trickle*,* less than a tsunami*,* of patients coming into the emergency department. And that slow trickle of COVID patients as they came in*,* was more than matched by an absence of other emergency department patients who were staying out. So*,* it was like this eerie experience of working in very quiet emergency departments knowing that we’re surrounded by this pandemic that’s*,* on some level*,* ravaging our nation.*

## Discussion

In this qualitative study of physicians’ experiences during the early pandemic, physicians described organizational, systemic, and interpersonal factors that served as barriers or facilitators to patient care and physician wellbeing. Frontline physicians, who provided direct patient care during the pandemic’s onset, were uniquely positioned to provide valuable insights into the challenges and opportunities provided by this and future pandemics.

Important facilitators to patient care identified at the organizational and systemic level included timely and clear communication across organizational levels and policies that supported nimble adaptation to rapidly changing circumstances. For example, removing administrative, financial and policy barriers to facilitate rapid adoption of telehealth visits and redeploying physicians to assist in different specialty areas allowed for continuing care while reducing the burden on physicians in specialties most impacted by COVID-19. Similar redeployment strategies were adopted by many health systems worldwide to facilitate patient care during the pandemic, with transparent communication, shared decision-making, and collaboration with frontline staff found to be key factors in the success of such adaptations [31]. State-level policies also play an important role in facilitating organizations’ ability to adapt. For example, community health centers in states with favorable telehealth reimbursement policies shifted substantially more visits to telehealth during the pandemic [32]. 

Despite some positive organizational and systemic adaptations, many challenges remained. Physicians in our study noted the financial pressures created by the loss of elective procedures in a volume-dependent reimbursement model. These pressures disproportionately impacted certain specialties and types of practices. Similarly, resources to facilitate patient care were unevenly distributed towards the acute phase of care, neglecting the needs of patients who required longer-term support as they recovered from COVID-19. This variation in adaptation resulted in varied experiences for both physicians and patients across regions and types of organizations. Identifying and addressing disparities in access to resources across settings during large-scale emergency responses is needed to ensure inclusive and equitable care.

The importance of aligning resources with the needs of frontline clinicians and patients is a critical lesson learned from the early pandemic. For example, some physicians in our study observed that the deployment of military field hospitals in their local region, while well-intentioned, did not prove useful because they were designed to support patients without COVID-19 during a time when the inpatient population was primarily driven by COVID-19 admissions. This mismatch suggests the need for more flexibility to adapt federal or state resources to meet local needs.

Some adaptations, such as the rapid adoption of telehealth, had both positive and negative aspects. On the one hand, the shift to telehealth enabled healthcare visits that may otherwise have been canceled, improved no-show rates in patient populations that typically have logistical barriers to attending in-person appointments, and protected healthcare workers and patients from exposure to COVID-19. On the other hand, physicians reported concerns that telehealth was not as accessible for rural and remote populations or those without reliable broadband internet access, and uncertainty around reimbursement made the decision to shift to telehealth more difficult for smaller practices. Although our study reports on telehealth perspectives from the early pandemic, subsequent studies suggest that potential disparities persist in access to and effectiveness of telehealth services based on factors such as geographic area, race/ethnicity, language, and socioeconomic status [33]. 

In addition to patient care challenges, physicians reported many stressors that made self-care difficult during the early pandemic. Acute stressors were often related to risks of infection to themselves, colleagues, and family members. Many physicians expressed feeling torn between the need to keep their unit/clinic running and the duty to protect potentially vulnerable staff. Physicians appreciated when their organizations took a proactive approach to protecting healthcare workers, such as early attention to PPE distribution and COVID-19 screening protocols. However, problems with access to testing and PPE during the pandemic were widespread, suggesting the need to improve our national supply chain and develop workflows for efficient distribution of supplies during times of emergency [34]. 

Both organizational and interpersonal support played an important role in physician wellbeing during this period of uncertainty. Physicians appreciated structured support from existing organizational wellness programs and opportunities to debrief. Although physician wellness programs are widely available within medical schools [35], physicians working outside of academic medicine may not have access to these important structural wellness supports. A majority of U.S. hospitals offer employee wellness programs, but these largely focus on promoting healthy nutrition and physical activity or offering preventive health screenings [36]. Physician-specific support programs may be beneficial to address the unique stressors and concerns of the physician workforce. A recent systematic review of interventions to improve physician wellness found that most successful interventions included some combination of peer support or mentorship with individual stress-management education [37]. Health systems could consider implementing similar initiatives in the absence of formal physician wellness programs, although more research is still needed to determine the optimal approaches to providing workplace support for physicians. Recent recommendations include using a trauma-informed care approach to assess and support frontline clinicians, which may be especially important during and after crises like the COVID-19 pandemic [38]. 

Outside of organizational wellness support, many participants in our study benefited from informal peer support as a means of exchanging stories and discussing challenges. In addition to taking comfort in shared experiences, participants noted that informal support from trusted peers provided an outlet to express feelings without worrying about potential professional repercussions. To expand opportunities for peer support beyond the pandemic, physician leaders could offer training to provide peer support and promote activities that provide informal socialization and camaraderie-building among colleagues. In addition, physician leaders can help to normalize help-seeking behaviors. Seeking formal help for stress or mental health concerns remains stigmatized among physicians, which is well documented in the literature [39]. Overcoming these barriers is essential to reduce burnout, improve clinician wellbeing, and ensure that frontline providers have the support to provide empathetic care to patients [40]. 

### Limitations

Our study has several limitations. Qualitative data from the relatively small number of physicians cannot be fully representative of facilitators and barriers. In fact, it is possible that clinicians who were available to participate in our study experienced more organizational support and fewer barriers compared with clinicians who were not available to participate. For feasibility we used a combination of individual interviews and focus groups. However, using two different formats may have elicited different responses. For example, participants from focus groups could have been influenced by other participants to discuss topics they would not have introduced on their own in an individual interview. The intentional sampling of what were identified as frontline physicians at the time limited perspectives from other specialists. Additionally, most participants were from urban areas. Including a broader range of physician perspectives or larger number of participants might have yielded different insights. The early time frame meant those we interviewed were in the raw period of extreme uncertainty and hadn’t had time to reflect with hindsight. While our study provides descriptive information about variation in pandemic responses across organizations and how those responses were perceived by clinicians, we did not collect detailed information about the characteristics of each participant’s setting and our questionnaire was designed to capture experiences broadly rather than to explore the impact of these characteristics, specifically. Future studies could expand on these findings by further exploring how regional and organizational characteristics impacted clinicians’ experiences and patient care.

## Conclusion

The lived experiences of physicians provide critical insights into how American health systems have responded and adapted to the COVID-19 pandemic. This study highlights ways in which, in the early pandemic, organizational and systemic factors impacted both patient care and self-care among physicians across specialties. Physician experiences offer guidance to health care organizations and policy makers as they take active steps to plan responses to future events.

## Electronic Supplementary Material

Below is the link to the electronic supplementary material.

Supplementary Material 1

Supplementary Material 2

## Data Availability

No datasets were generated or analysed during the current study.

## Abbreviations

ED: Emergency department
PPE: Personal protective equipment
