# Supplementary material for: Experiences of U.S. frontline physicians during the COVID-19 pandemic: a qualitative study
Source: Arch Public Health. 2025 May 7;83:122. doi: 10.1186/s13690-025-01609-0 (PMC12057006; doi:10.1186/s13690-025-01609-0)
Supplement: Supplementary file 1 — Supplementary Material 1 [file 13690_2025_1609_MOESM1_ESM.docx]

| Questions | Probes |
| --- | --- |
| 1. How would you describe what it is like practicing medicine during COVID-19? | - 1. *Probe:* How has this pandemic impacted you professionally and personally? |
| 1. What got you through the most difficult times? | - 1. *Probe:* Coping, social/emotional support, etc. |
| 1. What are your initial thoughts about the survey? | - 1. *Probe:* The topics? (telemedicine practice, PPE health impacts, professional fulfillment index)   2. *Probe: Are there other questions we could ask that measure overall health and well-being? How important are questions about social support, emotional/mental well-being, perceptions of physical health?*   3. *How do you think your colleagues would feel about discussing these topics?*   4. *Probe: Based on your professional experiences, what other topics or question should we include in the survey? (lessons learned, unmet needs, self-care, impact on job security, strategies used to protect your families from exposure, personally been sick)?*   5. *Probe: Based on your professional experiences, are there topics or questions that should not be included in the survey?*   6. *Probe:* Length? |
| 1. What do you think about the wording of the questions? | - 1. *Probe: What questions are unclear or confusing?*   2. *Probe: Are there questions that are not specific, or you are unsure about the purpose?*   3. *Probe: Comments on the number/type of open-ended questions? More or fewer?*   4. *Probe: Were there any questions that you think your colleagues may feel uncomfortable in answering?* |
| 1. *What do you think about the flow of the questions?* | - 1. *Probe: Logical order?* |
| 1. *Do you have any other thoughts about the survey and/or your experiences that you would like to share?* |  |
