# Supplementary material for: Experiences of U.S. frontline physicians during the COVID-19 pandemic: a qualitative study
Source: Arch Public Health. 2025 May 7;83:122. doi: 10.1186/s13690-025-01609-0 (PMC12057006; doi:10.1186/s13690-025-01609-0)
Supplement: Supplementary file 2 — Supplementary Material 2 [file 13690_2025_1609_MOESM2_ESM.docx]

**Supplementary Appendix Table A2.** (online only) Themes and Subthemes Related to Physician Experiences During the Early COVID-19 Pandemic in the United States

| **Theme #1 Facilitators to Patient Care & Physician Wellbeing** | |
| --- | --- |
| ***Subtheme*** | **Representative Quote (physician specialty)** |
| ***Subtheme 1A: Organizational Leadership and Operational Support***  Resource sharing and cooperation between departments, proactive management of frontline provider needs, and flexible adaptation of policies facilitated patient care and made physicians feel protected | The hospital did a lot of work to bring in other people to the Emergency Department …. I was working with Urologists and Ophthalmologists who had come in and we would train them for two shifts and then they would be attendings and second attendings. (Emergency Medicine)  They have spun up telemedicine programs with an amazing degree of innovation. They've ignored requirements and regulations that previously would kind of bind them. And fortunately, CMS has issued waivers and the Joint Commission has stopped inspections. And so hospitals are more willing to innovate and do things. (Emergency Medicine)  They fitted us for N-95s, and they used the hospital screening protocols and all that good stuff right from the outset, so that definitely felt safer. (Primary Care)  We have issues around transportation…we have a vulnerable population. We also have a lot of construction going on and lack of parking, so [with telehealth appointments] our no-show rates had plummeted. When people were actually scheduling these virtual or telephone visits, they were more likely to keep them because, one, they have nothing else to do, they’re at home, and, two, they don’t have the challenges of getting to the clinic. (Primary Care)  Fortunately, the hospital… leadership had actually done quite good with, planning in advance in terms of making resources available as well as in terms of actually opening a field hospital in order to really manage the patient population and I think similar adjustments were being done even in the ICU setting. (Critical Care) |
| ***Subtheme 1B: Organizational protection of physician self-care and wellness***  The availability of institutional wellness programs facilitated healthy coping and wellbeing. | I think our program in particular has a very strong wellness program and so they were very active in rolling out a lot of immediate issues that they made, we all had access to free video counseling. (Emergency Medicine)  The institution has been very supportive … there's a system wide debriefing as well as a division level debriefing that has occurred and [is] very helpful… and overall from a professional perspective it's been a very supportive environment. (Hospitalist)  ACEP, the American College of Emergency Physicians that I also work with has also put together a video diary or testimonial that is private that allows people to discuss their, their feelings, their anxieties, their lessons learned that they would like to share, but in a way that's private and not put out on social media. (Emergency Medicine) |
| ***Subtheme 1C: Peer and Family Support/ Debriefing***  Support from colleagues and family members through conversation serves an important role in upkeeping morale and mental health. | I find the most relief and solace when we can get together for a virtual happy hour with others in our group. The question is how can we do a better job at helping prepare and deal with pandemics? The most benefit is probably in doing more peer counseling. There is still a lot of reluctance among physicians to seek formal help. We all hear those cases where someone tried to help a peer, reported it to the medical board and that person lost their license. (Emergency Medicine)  In terms of dealing with it…I just try to talk it out with another colleague and let it go by (Critical Care)  I think where we turn to that’s healthy is turning to our peers for some peer support. And equipping our peers to better deal with that, I think, is really important. (Emergency Medicine)  And then as you realize how much it's affected your professional and personal life, so having that behavioralist actually around to support all of us has been tremendously helpful, again in normalizing some of the reactions that we have. That's been huge. My fellow faculty, we spend a lot of time together and I think actually in isolation, we're spending more time together because they're the only people I talk to other than my family…we're actually spending more time together, masked up and 10 feet apart, we talk through our office walls often. But that's been very helpful to know that there's a cadre of others who are going through the same things, both at work and at home. (Primary Care)  The main things that are helping me cope are good family connections. So having, maintaining really close relationship and open communication with my immediate family. And then there's been a lot of reconnection with past social networks that I hadn't really connected with as much with recently. So, especially people who are going through similar things. So both with my med student, med school friends, as well as my residency friends. There's been reconnection, whereas I don't know that we would have talked as much had this never ended up happening. (Primary Care)  Our clinicians really want to help. They really want to step up and rise to this moment. So early on, I will admit some of us was pretty scared. We were thinking, I know we signed up for this sorta, but, I prefer not to die from COVID, or really have it. But then I also saw some people who just said, let's just one foot front of the other, we're just going to do it. We're just going to make new systems and processes. We're going to get through this, we're going to level, we're going to go together. We're going to huddle every morning, every afternoon. We're gonna figure out new ways, no whining or if you whine it's okay. But, it's only the let off some steam and we're, we're gonna, we're going to do this. (Primary Care)  What's been really helpful to me personally is to know that these... this cycle or this path of milestones that occurs during disasters has been described before in the literature and actually what I'm going through is what is expected, which is this curve of having a lot of optimism in the beginning and almost like a heroicism. And then followed by a period of prolonged kind of disillusionment as you're trying to work through all the workarounds. (Primary Care) |
| **Theme #2: Barriers to Patient Care & Physician Wellbeing** | |
| ***Subtheme*** | **Representative Quote (Physician Specialty)** |
| ***Subtheme 2A: Bureaucratic Difficulties, Lack of Standardization in Governmental Guidelines and Organizational Support***  Lack of consistent organizational and governmental support or misalignment of supports with frontline needs created challenges | There was definitely a very noticeable lack in coordination in leadership. I mean I think obviously on the federal level. But even on a local level it was challenging. I work for the largest health system in the city and we have about 11 different hospitals across the city. And within our health system, the burden of disease and the onset of this, the onslaught of patients were very different (Emergency Medicine)  It is very hard because what you would like to do and what policy would allow us to do are very different. Even when the military stepped in and started directing field hospitals, the purpose of the field hospitals was never to offload COVID patients from any hospital in the entire county…they were intended for non-COVID patients. But we were seeing a dramatic decline in non-COVID hospitalizations around the same time that COVID was booming around the country. (Hospitalist)  Until recently we couldn't charge for telemedicine visits…there's very limited things that you can bill for telehealth. And so, if people couldn't get compensation from it, they were much less likely to implement it. But now, at least from the COVID perspective, we've had an emergency thing put in at least temporarily. (Emergency Medicine)  In many rural areas of Texas where I am, we've talked about trying to help our rural hospitals that are failing, but they just don't have the internet infrastructure in place to be able to even do telemedicine in a lot of places. (Emergency Medicine)  My practice in the beginning said, "We don't care if we get paid or not, we're part of a large institution.” We have a very vulnerable population. Many of them will not have the ability to use video or just not have the data minutes to do that. If we were a private practice and before the Medicare retroactively allowed for billing for the telephone visits, we would have had a very different conversation around how to keep potentially volumes either up or... and that was not an issue for us. (Primary Care)  If you are in an academic medical center you may be a little bit more sheltered from the financial pressures than a partnership is where your financial income really is dependent directly on your volume. (Emergency Medicine)  We don't have a capacity to handle these many patients for the recovery phase for the rehab and so on. So, that's also another external factor. Either insurance is going to change the reimbursement, or a health care policy have to give them more support for rehab- to accept these patients so that they can get the care to recover, because I do believe the reason we are doing- providing this level of care is for them to recover and not to, just to prevent the deaths… I feel like system is failing because we don't have a support for the recovery phase. We got a lot of support for the acute phase. (Critical Care) |
| ***Subtheme 2B: Uncertainty resulting from poor communication or lack of preparedness***  Rapidly changing information and lack of clear protocols can impede a physician’s workflow. | The way that information was communicated or has been communicated or even currently is communicated through the usual channels was altered because our understanding of the virus changed so much daily. So that was frustrating, but also actually pretty scary… but what has happened and is continuing to happen is that we're getting our sources of information from so many different places that we usually wouldn't. (Primary Care)  So how strict the criteria were the first two weeks, I really couldn't get [a COVID test] unless if someone was sick enough to get in the hospital. There were barriers. We just didn't have enough tests. There was a real gatekeeper approach from our organization. (Primary Care)  I think it's scattered. It feels like we don't know the principles behind what we need to be doing and- and I think because of that it's hard to figure out what we should be doing and figure out what our organizations should be doing to support us. (Primary Care)  Part of the really fascinating thing about this is health care practice, policy, implementation, not to mention the evidence used to come on a monthly, quarterly basis. Now it's coming to two to three times a day. When you think about that work speed change and stuff and often it's based on very little data. It's just, we're going to do it this way. And then you try and systematize it and learn from others. (Primary Care)  So, for me to get information on COVID from the New York Times would be very unusual but having the pressure of trying to get as much information as possible, not necessarily trusting the source of information and then having to answer questions from patients from information that had not really been vetted in a way that typical information would have been vetted. In this case the best I could do might be the CDC website which was behind and also I’d say the information that I was receiving as a physician was colored or influenced by the availability of the test. That was a problem. (Primary Care)  So for me to make patient care decision based on some email that a hospitalist showed me on her phone, that's not the way that information is usually communicated or disseminated in medicine. So essentially looking at case reports and case series that aren't even vetted in peer-reviewed journals was very disconcerting. So I both wanted to work, but also had a lot of trepidation around working because of the uncertainty (Primary Care) |
| ***Subtheme 2C: Interpersonal barriers to physician wellbeing***  Physicians’ personal lives and professional goals were impacted by the pandemic, causing additional stress beyond navigating the challenges of patient care | I would say that the first few weeks, I think we were all... I'll just speak for myself, was probably more optimistic and felt that it was likely a time-limited change in practice. But I think as the weeks have progressed, it's actually gotten harder, not medically but actually personally has gotten harder to balance working full time, managing a household with children, school-age children (Primary Care)  I think the other piece that people who are building things, like let's say QI initiatives in their clinic or research programs is this idea that you have dedicated your career or your professional life to projects that now have become derailed.(primary care)  I think there's still a lot of reluctance amongst physicians for both good and bad reasons to seek formal help…we all hear that one crazy case of that person who tried to do the right thing and, and address their depression and stress and instead things went haywire and they lost their license when they got reported to the medical board, right? So I think there's always fear about that issue (Emergency Medicine) |
| **Theme #3 Acute Stressors** | |
| ***Subtheme*** | **Representative Quote (Physician Specialty)** |
| ***Subtheme 3A: Concern about Exposure***  Significant anxiety and stress due to the possibility of infecting one’s self and family often resulting from a lack of PPE. | And then now am I coming home and infecting my own family members? Am I contaminating my entire house? (Emergency Medicine)  Having a sense of responsibility to the people who work with us. So, our MAs, our nurses, our support staff and the idea of not being able to protect them in the way that we feel as doctors should be happening… and I know that this employee is pregnant or has asthma or this employee has a heart condition and I am supposed to tell them all to keep coming to work… that sense of going against what clinically you feel is rights and what the health system needs to function. (Primary Care)  I tried to go outside… to the park, to walk around before I go to ICU. I do start preparing probably two days prior. I go to the ICU just to keep myself in correct mode because I have to take care of myself, but I also have to take care of a resident and the nursing staff and patient and patient families. So, I really have to keep myself neutral and strong…so that's a lot. (Critical Care)  My wife has had rheumatoid arthritis for 40 years. I told the team, I am happy to do in-patient work. I'm happy to do outpatient work. Uh, I'm going to choose not to do the COVID team because I ... while I think I would do okay even if I got it, I don't need my wife to get it. (Primary Care) |
| ***Subtheme 3B: Feeling Unprepared for New Clinical Challenges***  Lack of information, supplies contributed to acute stress and feeling unprepared to fight the COVID-19 pandemic. | And we honestly didn't know how to answer a lot of the questions in the beginning, so that was a great... a big source of stress was that if a patient emailed me, I wouldn't necessarily know even how to answer them (Primary Care)  Usually, physicians are in the place of being the authoritative figures in terms of answering medical questions and to not have that information, or to have information I was questioning as suspect was very troubling. And that's for patients. I think the added thing for an academic is that residents often come and ask for supervision and ask questions and to not be able to answer those questions or again, to answer them in a way with such limited information and have to make patient decisions about management was very...disconcerting (Primary Care)  As an ER doctor I am accustomed to making decisions with incomplete information but in this case with the stakes being particularly high it’s very stressful to be looked to as the person who has the answer when no answers exist. (Emergency Medicine)  I would say there's a lot of uncertainty about what to recommend to people. I feel like things are constantly changing. I think anxiety is a little bit higher… I would go back and say that uncertainty is related both to clinical things as well as workflow and administrative tasks. (Primary Care) |
| ***Subtheme 3C: Anticipating the Worst***  Anxiety and stress derived from anticipation of worst-case scenarios including an influx of COVID-19 patients. | We were not hit hard by COVID and kept waiting for things to get worse, which created anxiety and fear. The uncertainty of how bad things would get and when was stress-producing (Emergency Medicine)  I kind of always felt like, I don't know if anyone's played sport but, you get kind of nervous before a match and then the match gets delayed. And gets delayed again and you're just in a state of nerves all the time (Emergency Medicine)    From an Emergency Department perspective, I think there was a preparation for an onslaught. We're so used to disasters being presented as this surge of sudden onrushes of patients. So, we were really preparing for that at least across the West Coast and [at our] sites. And really what we found was more of this slow trickle, less than a tsunami, of patients coming into the Emergency Department. And that slow trickle of COVID patients as they came in, was, more than matched by an absence of other Emergency Department patients who were staying out. So, it was this eerie experience of working in very quiet Emergency Departments knowing that we're surrounded by this pandemic that's on some level, ravaging our nation. (Emergency Medicine) |
